# Catheter-Directed Thrombectomy for Pulmonary Embolism in the Setting of Acute Stroke

**DOI:** 10.7759/cureus.9874

**Published:** 2020-08-19

**Authors:** Clay H Hoster, Michael J Herring, Maria Isabel C Planek, Steve Attanasio

**Affiliations:** 1 Internal Medicine, Rush University Medical Center, Chicago, USA; 2 Cardiovascular Medicine, Rush University Medical Center, Chicago, USA

**Keywords:** pulmonary embolism, thrombectomy, echocardiography, pulmonary angiography, cerebrovascular accident, catheter-directed thrombolysis, deep vein thrombosis (dvt)

## Abstract

A 79-year-old male presented with an acute stroke and was treated with tissue plasminogen activator (tPA). His neurological symptoms improved, but he subsequently developed hemodynamic instability requiring intubation and vasopressors. Imaging studies revealed a massive pulmonary embolism as the cause of his worsening clinical picture. Mechanical thrombectomy using traditional devices was deemed too risky as the patient could not safely tolerate the usual anticoagulation dosage these devices require. The Penumbra Indigo® system (Alameda, CA, USA) was thus chosen for its ability to achieve thrombus aspiration within a lower therapeutic heparin range. Pulmonary artery aspiration thrombectomy was done using the device, and three days after the procedure, he was extubated and weaned completely off vasopressors. The therapy’s efficacy despite the patient’s unique life-threatening conditions demonstrates a novel application of the state-of-the-art pulmonary embolism treatment currently in research.

## Introduction

Pulmonary embolism (PE) is the third-leading cause of cardiovascular-related death in the United States, accounting for around 100,000 deaths per year [1]. Specifically, massive (or high-risk) PE, defined as acute PE with sustained hypotension and hemodynamic instability, accounts for an estimated 4.5%-10% of all PE cases and has over 50% mortality [2,3]. Historically, the primary treatment options for PE have been anticoagulation therapy and systemic thrombolytic therapy. However, both treatments carry with them associated increased risks of bleeding. Catheter-directed therapies for PE can remove obstructive emboli directly and allow for rapid patient improvement. As a result, catheter-directed therapies have become more favorable over the last two decades [3]. We describe a patient with acute stroke who developed hemodynamic instability from a massive PE with resolution using catheter-directed thrombectomy.

## Case presentation

A 79-year-old male patient with a past medical history of hypertension, hyperlipidemia, type II diabetes, and a previous deep venous thrombosis (DVT) experienced a syncopal episode while at home. In the emergency room of an outside hospital, he was found to have right-sided weakness, gaze deviation, and altered mental status consistent with a left middle cerebral artery stroke. A CT scan of his head was negative for a bleed. The patient was administered tissue plasminogen activator (tPA) 173 minutes after he was last known to be without symptoms. Following the treatment, his neurological symptoms improved. A subsequent CT angiogram of the patient’s head and neck showed normal cerebral vasculature without occlusive thrombus or dissection. After his stroke symptoms largely abated, the patient developed respiratory distress. He was intubated and started on vasopressors, but he remained persistently hypoxic. Initially, the patient was believed to have aspirated. As his clinical picture worsened, the differential was broadened to include septic, cardiogenic, and obstructive shock. The patient’s history of a DVT suggested a potential massive PE which could result in obstructive shock. His prior DVT occurred over 10 years ago, and he completed an unspecified anticoagulation regimen at that time. The patient was transferred to Rush University Medical Center (RUMC) for escalation of care.

**Figure 1:**
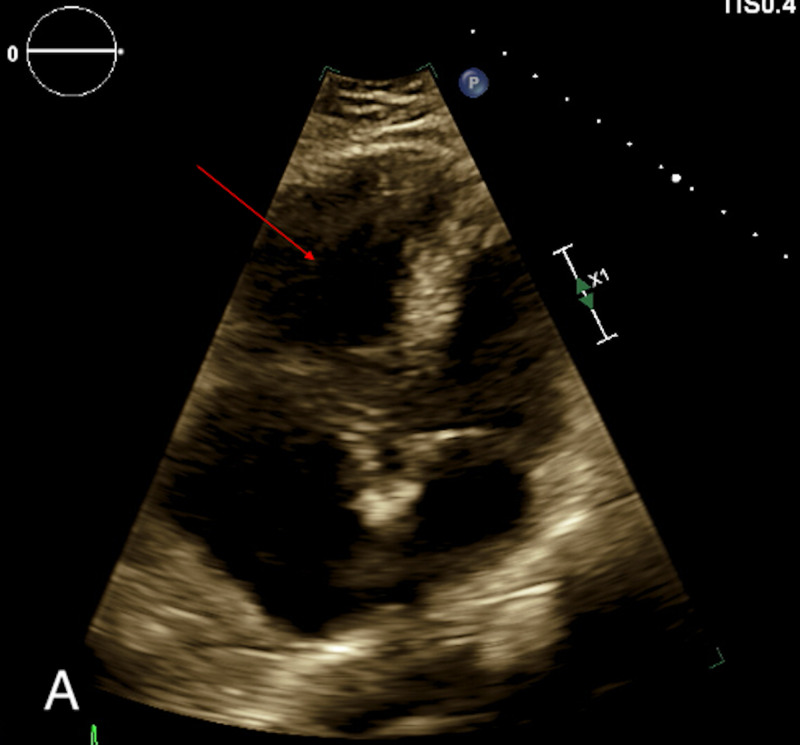
Initial transthoracic echocardiography of the patient described above. The right ventricle is severely dilated showing McConnell’s sign (red arrow).

**Figure 2:**
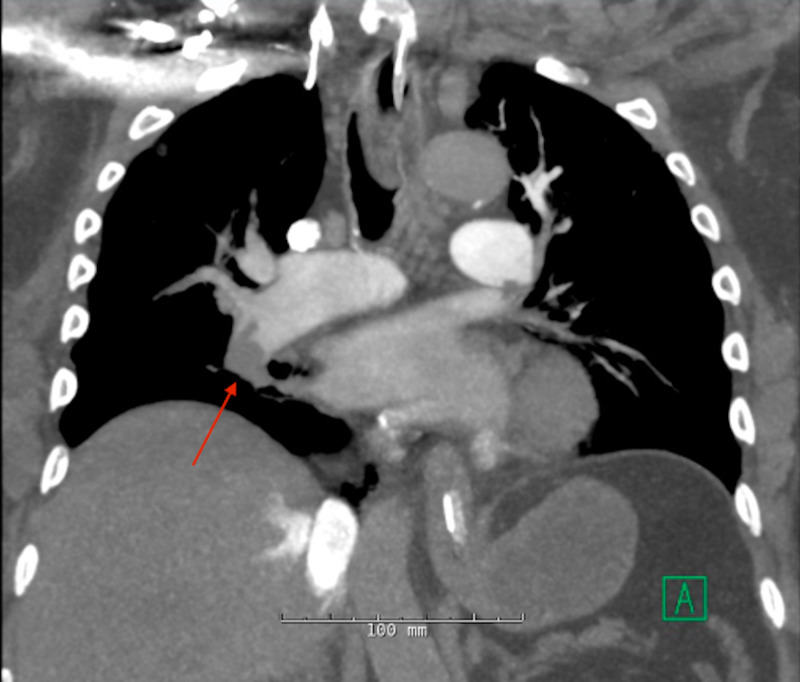
Initial CT angiography of the lungs in the setting of hypoxia shows a large clot burden in the right pulmonary artery (red arrow).

A transthoracic echocardiogram (TTE) showed a severely dilated right ventricle consistent with McConnell’s sign (Figure 1). Lower extremity ultrasound showed an extensive DVT in the right leg. These findings strongly suggested a PE as the cause for the patient’s instability. A subsequent CT pulmonary angiogram confirmed this suspicion by revealing a large clot burden in the right pulmonary artery (Figure 2). It also showed chronic emboli in the left main pulmonary artery. The patient was started on a heparin infusion within 24 hours of receiving tPA. Once the heparin was at a therapeutic level, a repeat head CT was obtained and, again, was negative for bleeding. Despite initiation of heparin, he remained hypoxic and hypotensive.

The patient developed new-onset atrial fibrillation after arriving at RUMC. This arrhythmia likely resulted from the increased demand on the heart from the extensive clot burden in the lungs. The patient experienced multiple episodes of rapid ventricular response that were unsuccessfully treated with digoxin. He was started on an amiodarone infusion that better controlled the rate.

To resolve the PE, mechanical thrombectomy was considered, but at the advice of the stroke team, it was deemed too risky; the patient could not safely tolerate the usual therapeutic dosage of heparin needed to perform the procedure with the traditional devices. Thus, the Penumbra Indigo® system (Alameda, CA, USA).was chosen for its ability to achieve thrombus aspiration within a lower therapeutic heparin range. Additionally, the device was of a small enough caliber to operate within the pulmonary arteries.

**Figure 3:**
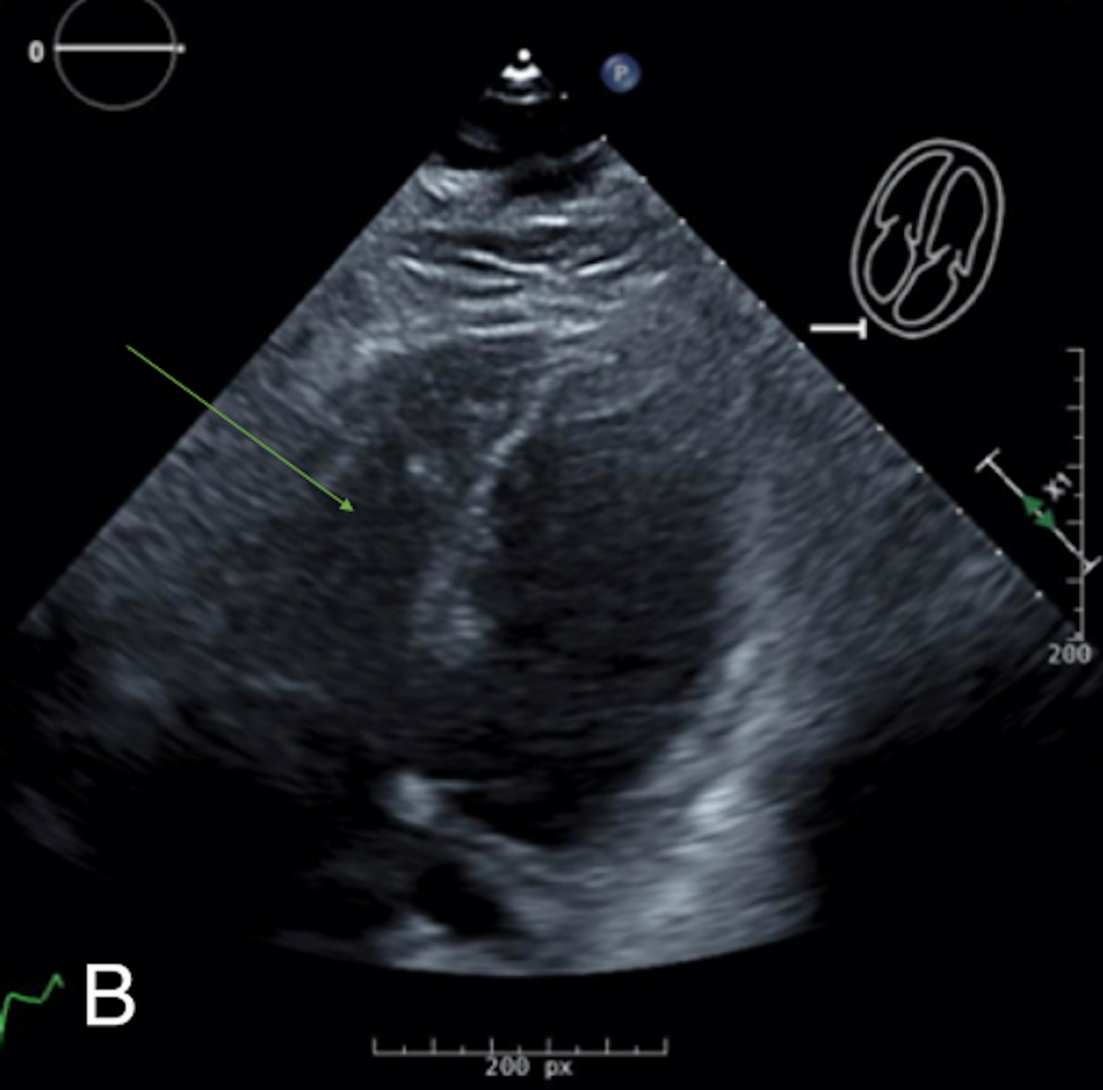
Repeat transthoracic echocardiogram one day after catheter-directed therapy shows drastic improvement in the size of the right ventricle (green arrow).

Percutaneous pulmonary artery aspiration thrombectomy was done via the right internal jugular vein using the Penumbra device. An inferior vena cava (IVC) filter was also placed during the procedure. By the next day, TTE showed that the patient’s right ventricle had already significantly decreased in size (Figure 3). Three days following the procedure, he was extubated and weaned completely off vasopressors.

As the patient continued to improve, a transesophageal echocardiogram (TEE) was obtained that showed evidence of a patent foramen ovale (PFO). No evidence of left atrial appendage thrombus was seen on the TEE. Direct current cardioversion (DCCV) was done to treat his atrial fibrillation, and he was successfully converted into sinus rhythm. In an attempt to determine the cause of the DVT, a CT scan of his abdomen and pelvis was obtained. The imaging did not reveal any specific malignant or metastatic processes to explain his hypercoagulable state. Further investigation into his DVT was deferred as the findings would not change his coagulation management: life-long anticoagulation therapy. Prior to discharge, the patient was taken off of the heparin infusion and he was started on oral apixaban to be taken indefinitely.

Ultimately, the etiology of the patient’s stroke was not determined. One potential etiology was a cardioembolic stroke brought on by the new-onset atrial fibrillation. Alternatively, the stroke could have been due to a venous thromboembolism which traveled through his pre-existing PFO. 

## Discussion

Catheter-directed therapy is currently indicated to treat patients with massive PE who have contraindications to systemic thrombolysis or who have failed previous treatments and remain unstable. While not currently indicated for the treatment of submassive PE, catheter-directed therapy has been used to treat submassive PE more frequently in recent years. This selection is driven by the therapy’s potential to decrease some of the long-term effects of PE such as the development of pulmonary hypertension [3]. 

There are a number of catheter-directed therapies available to achieve the ultimate goal of thrombus removal, each with differing risks. Ultrasound-assisted thrombolysis uses high-frequency sound waves to penetrate the clot. However, benefit has only been seen when used in conjunction with thrombolytics [4]. Rheolytic thrombolysis uses pressurized saline to break up the clot, while the clot’s fragments are aspirated through the catheter. Issues with this technique include the need for a large catheter, the release of adenosine from the disrupted platelets, and the potential for hemoglobinuria from broken-down red blood cells [5]. Rotational thrombolysis is a similar technique to rheolytic thrombolysis, except that it uses a rotating device at the tip of the catheter to break up the clot for removal. Suction thrombectomy uses a catheter to aspirate the entire thrombus, typically without the administration of local thrombolytic therapy.

**Figure 4:**
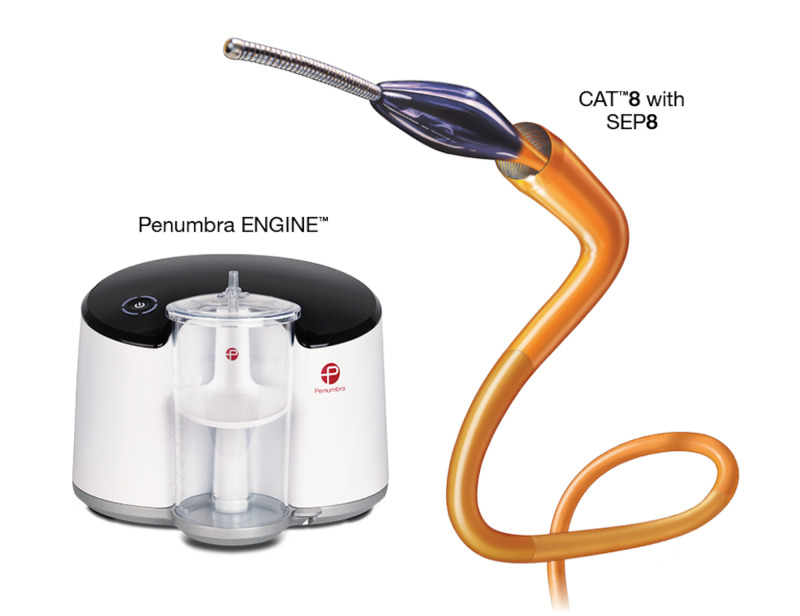
Picture of the Penumbra Indigo® Aspiration System showing the CAT™8, SEP™8, and ENGINE™. The CAT™8 (size: 8-French/2.67 mm diameter, 85 cm length) is the suction catheter used to remove the clot. The separator (SEP™8) allows the user to macerate the clot if necessary while aspirating it through the lumen of the CAT™8. The ENGINE™ powers the vacuum and collects the aspirated clots. Image reproduced with permission from Penumbra Inc. (Alameda, CA, USA).

The numerous devices utilized for suction embolectomy vary slightly in their approaches to achieve thrombus aspiration. The 20-French catheter FlowTriever® system (Inari Medical, Irvine CA, USA) was studied in the FLARE (FlowTriever Pulmonary Embolectomy Clinical Study) trial published in 2019. Patients with acute PE saw improvement in right ventricle/left ventricle (RV/LV) ratio with only one patient experiencing a major bleeding episode. Approximately 98.1% of the cases used no adjunctive thrombolytics [6]. The AngioVac® system (Angiodynamics Inc., Latham, NY, USA) utilizes a 22-French catheter to remove emboli. This device has seen very limited use in PE treatment because its large caliber makes it difficult to reach the pulmonary arteries [7]. The Penumbra Indigo system (Figure 4), as used in our case, utilizes a smaller caliber 8-French catheter for clot removal. This device was first used for patients with embolic stroke; however, the ongoing EXTRACT-PE (Evaluating the Safety and Efficacy of the Indigo Aspiration System in Acute Submassive Pulmonary Embolism) trial has demonstrated promising results for patients with acute PE [8]. The primary endpoint of the trial was met with a significant mean reduction in RV/LV ratio of 27.3% after 48 hours of the intervention. Most importantly, as it pertains to our case, no thrombolytic drugs were used in 98.3% of cases, making it a very promising tool for patients with increased bleeding risks. Adverse events were rare, with one instance of bleeding from the groin access site and one death 11 hours post-procedure due to sustained ventricular tachycardia (Clinical trial: Sista A. EXTRACT-PE; 2017-2020).

## Conclusions

Systemic thrombolytic therapy is typically indicated for hemodynamically unstable patients with pulmonary emboli. However, as seen in our case, catheter-directed thrombus removal can be used in patients who are at a high risk of bleeding or who have already received systemic therapy and remain unstable. Furthermore, our case describes a unique utilization of catheter-directed therapy in the presence of acute stroke: a novel application of the state-of-the-art catheter-directed PE treatment currently in research. We suggest using catheter-directed techniques on a case-by-case basis with special consideration in patients who are unable to receive further anticoagulation.
